# The prognostic value of admission lymphocyte-to-monocyte ratio in critically ill patients with acute myocardial infarction

**DOI:** 10.1186/s12872-022-02745-z

**Published:** 2022-07-07

**Authors:** Yuanyuan Zhao, Chunshu Hao, Xiangwei Bo, Zhengri Lu, Hao Qian, Lijuan Chen

**Affiliations:** 1 Department of Cardiology, Zhongda Hospital, Southeast University, Nanjing, China; 2 School of Medicine, Southeast University, Nanjing, China; 3 Department of Cardiology, Nanjing Lishui People’s Hospital, Zhongda Hospital Lishui Branch, Southeast University, Nanjing, China

**Keywords:** Lymphocyte-to-monocyte ratio (LMR), Acute myocardial infarction (AMI), Propensity score matching, Mortality, Intensive care unit (ICU)

## Abstract

**Background:**

Inflammation plays a critical role in acute myocardial infarction (AMI). Recent studies have shown the value of hematologic indicators in MI risk stratification and prognostic assessment. However, the association between lymphocyte-to-monocyte ratio (LMR) and the long-term mortality of critically ill MI patients remains unclear.

**Methods:**

Clinical data were extracted from the Medical Information Mart for Intensive Care III database. Patients diagnosed with AMI on admission in the intensive care units were include. The optimal cutoff value of LMR was determined by X-tile software. The Cox proportional hazard model was applied for the identification of independent prognostic factors of 1-year mortality and survival curves were estimated using the Kaplan–Meier method. In order to reduce selection bias, a 1:1 propensity score matching (PSM) method was performed.

**Results:**

A total of 1517 AMI patients were included in this study. The cutoff value for 1-year mortality of LMR determined by X-Tile software was 3.00. A total of 534 pairs of patients were matched after PSM. Multivariate analysis (HR = 1.369, 95%CI 1.110–1.687, *P* = 0.003) and PSM subgroups (HR = 1.299, 95%CI 1.032–1.634, *P* = 0.026) showed that 1-year mortality was significantly higher in patients with LMR < 3.00 than patients with LMR ≥ 3.00 in Cox proportional hazard models. The survival curves showed that patients with LMR < 3.00 had a significantly lower 1-year survival rate before (63.83 vs. 81.03%, Log rank *P* < 0.001) and after PSM (68.13 vs. 74.22%, Log rank *P* = 0.041).

**Conclusion:**

In this retrospective cohort analysis, we demonstrated that a low admission LMR (< 3.00) was associated with a higher risk of 1-year mortality in critically ill patients with AMI.

**Supplementary Information:**

The online version contains supplementary material available at 10.1186/s12872-022-02745-z.

## Introduction

Cardiovascular diseases (CVDs) are the leading cause of global mortality and disability, bringing a great burden of disease to health expenditure [1]. The prevalence and death rate of ischemic heart disease (IHD) remain increasing during last decades [2]. There is an urgent need for health system to improve pre- and in-hospital care for acute coronary syndrome, so it appears to be particularly significant to identify patients at high risk for adverse outcomes of IHD.

The inflammation following acute myocardial infarction (AMI) plays a critical role in determining MI size and subsequent left ventricular remodeling [3, 4]. Immune cells such as neutrophils, monocytes/macrophages and lymphocytes are activated or recruited to the infarct area contributing to necrotic substance removal and tissue repair [5–7], which are considered new targets for myocardial protection [8] and prognostic prediction [9–11].

Hematological indices such as hemoglobin levels, serum albumin, white blood cells (WBC), platelet to lymphocyte ratio (PLR) and neutrophil to lymphocyte ratio (NLR) have gained attention because of their low cost and clinical accessibility, which has also been proved valuable for risk stratification and prognosis in IHD patients [12–18]. Lymphocyte to monocyte ratio (LMR), a novel predictor of inflammation, are concerned to be associated with the severity and outcomes of cardiovascular diseases [19–21]. Whereas, no researches demonstrate the relevance between LMR and long-term mortality of critically ill patients with AMI.

In this study, we aimed to determine the association between admission LMR and risk of long-term mortality in critically ill patients with AMI based on the Medical Information Mart for Intensive Care-III (MIMIC-III) database.

## Methods

### Data source

All the relevant data were obtained from the Medical Information Mart for Intensive Care-III (MIMIC-III) database (version 1.4). MIMIC-III is a freely available database containing the records of 46,520 critically ill patients admitted to intensive care units (ICUs) of the Beth Israel Deaconess Medical Center (Boston, Massachusetts) from 2001 to 2012 [22, 23], which contains dates of death up to 4 years. The establishment of the MIMIC-III database was approved by the Institutional Review Boards (IRB) of the Massachusetts Institute of Technology (MIT, Cambridge, MA, USA) and Beth Israel Deaconess Medical Center. The database documents included charted events such as demographics data, laboratory tests, vital signs, survival data and diagnostic information such as the International Classification of Diseases, Ninth Revision (ICD-9). We completed the National Institutes of Health online course and passed the exam named “Protecting Human Research Participants” (Record ID 36,331,340). The study was conducted in accordance with the Declaration of Helsinki (as revised in 2013).

### Population selection

We included all critically ill patients diagnosed with AMI using ICD-9 diagnosis codes at first ICU admission in MIMIC-III database. The exclusion criteria were as follows: (1) age less than 18 years old; (2) missing lymphocyte and monocyte counts values at first 24 h of admission.

### Data extraction

All data were extracted from MIMIC-III database using structure query language (SQL) with PostgreSQL (version 9.6). The code that supports the MIMIC-III documentation and website is publicly available, and contributions from the community of users are encouraged (https://github.com/MIT-LCP/mimic-website). The extracted data included: (1) demographics: age, gender, ethnicity and body mass index (BMI); (2) vital signs: heart rate (HR), systolic blood pressure (SBP), diastolic blood pressure (DBP), respiratory rate (RR), temperature and percutaneous oxygen saturation (SpO_2_); (3) comorbidities: congestive heart failure (CHF), cardiac arrhythmias, hypertension, diabetes, chronic pulmonary disease, renal failure, liver disease, coagulopathy and elixhauser comorbidity index (ECI); (4) laboratory parameters: peripheral white blood cell count (WBC), neutrophil count, monocyte count, lymphocyte count, platelet count (PLT), hemoglobin (Hb), hematocrit (HCT), glucose (Glu), blood urea nitrogen (BUN), serum creatinine (Scr) and LMR; (5) scoring system: systemic inflammatory response syndrome (SIRS), simplified acute physiology score (SAPS) and sequential organ failure assessment (SOFA); (6) treatment information: percutaneous coronary intervention (PCI) and coronary bypass artery grafting (CABG); (7) outcomes: ICU length of stay, hospital length of stay, in-hospital mortality, 30-day mortality and 1-year mortality. Variables with less than 30% missing values were imputed using the multiple imputation method.

### Statistical analysis

Continuous variables were presented as the mean ± SD or median (interquartile range) and compared by t-test or Mann–Whitney U test. Categorical data were presented as frequencies with percentages and analyzed by χ^2^ test. Skewness/Kurtosis test and histogram were adopted to assess the normality of variables. After propensity score matching analysis, the paired t-test and Wilcoxon rank sum test for continuous data and the McNemar test for categorical data was used for assessing the comparability of baseline characteristics in the matched groups. The optimal cutoff value of the LMR for 1-year mortality was determined by X-tile (Version 3.6.1, Yale University School of medicine) software [24]. The principle of the software is to enumerate continuous variables as cutoff values and log-rank tests were performed for all cases based on survival data separately, with the variable value corresponding to the smallest P-value being determined as the optimal cutoff value. Survival curves were estimated using the Kaplan–Meier method and compared by the log-rank test.

To reduce the selection bias between different LMR groups, propensity score matching analysis (PSM) was performed. The propensity score was calculated according to the following baseline characteristics: age, gender, congestive heart failure, hypertension, chronic pulmonary disease, renal failure, SpO_2_, WBC, Glu, Scr, BUN, SAPSII and SOFA scores. The psmatch2 package in STATA software was used to create matched sample. Propensity scores were estimated using logistic regression models. Patients were derived using 1:1 matching with a caliper of 0.02 and without replacement. A total of 1086 patients were propensity score-matched eventually.

The Cox proportional hazard model was applied for the univariate and multivariate analyses to identify independent prognostic factors of 1-year mortality. To evaluate the association between the LMR and mortality, model I was adjusted for age, gender and ethnicity; model II was adjusted for variables with *P* values less than 0.05 in the univariate regression analysis. The results are presented as hazard ratios (HR) and 95% confidence intervals (CI). Subgroup analysis were performed with Cox regression model according to age, gender, ethnicity, hypertension, CHF, cardiac arrhythmias, chronic pulmonary disease, renal failure, coagulopathy, PCI, CABG, SIRS, SAPS II, SOFA, HR, DBP, RR, SpO_2_, WBC, Hb, PLT, Glu, Scr and BUN. All tests were two-sided, and *P* values < 0.05 were considered significant. All statistical analyses in our study were performed using STATA V.16.0 and R version 4.1.0.

## Results

### Patient characteristics

A total of 1517 acute myocardial infarction patients were included in our study (Additional file 1: Fig. S1). The optimal cutoff value of admission LMR for 1-year mortality was 3.00 (with a sensitivity of 60.45% and a specificity of 62.06%) calculated by the X-tile software. Patients were divided into two groups according to the LMR: the low LMR group (LMR < 3.00, n = 647) and high LMR group (LMR ≥ 3.00, n = 870). The comparison of baseline characteristics between two LMR groups was summarized in Table 1. Before propensity score matching, there were significant differences in baseline data between the two groups. The low LMR group patients tended to be older with a lower DBP, SpO_2_ and higher HR, RR, WBC, PLT Glu, Scr, BUN, ECI, SIRS score, SOFA score and SAPSII score. Furthermore, patients with lower LMR had higher incidence of CHF, chronic pulmonary disease and renal failure while had a lower prevalence of hypertension and lower PCI or CABG treatment rate. With the use of propensity score matching (1:1 ratio), 543 pairs of patients were generated. The imbalance between patients with an LMR < 3.00 and an LMR ≥ 3.00 was significantly reduced (Additional file 1: Fig. S2), and almost all baseline characteristics were comparable between the two groups (Table 1).

**Table 1:** Baseline characteristics before and after PSM matched

Characteristics	Before PSM			After PSM		
	LMR < 3.00 (n = 647)	LMR ≥ 3.00 (n = 870)	*P* value	LMR < 3.00 (n = 543)	LMR ≥ 3.00 (n = 543)	*P* value
*Demographics*						
Age, years	71.14 (61.20–80.86)	66.54 (56.60–78.66)	< 0.001	70.48 (59.99–80.26)	72.08 (60.13–81.79)	0.269
Gender, male	415 (64.14%)	542 (62.30%)	0.462	348 (64.09%)	346 (63.72%)	0.899
Ethnicity, white	423 (65.38%)	584 (67.13%)	0.476	354 (65.19%)	381 (70.17%)	0.080
BMI, Kg/m^2^	27.16 ± 6.19	27.55 ± 5.77	0.205	26.94 ± 5.96	27.30 ± 5.85	0.297
*Comorbidities*						
Hypertension	274 (42.35%)	434 (49.89%)	0.004	246 (45.30%)	264 (48.62%)	0.274
Diabetes	173 (26.74%)	246 (28.28%)	0.508	134 (24.68%)	176 (32.41%)	0.005
Congestive heart failure	95 (14.68%)	59 (6.78%)	< 0.001	58 (10.68%)	56 (10.31%)	0.843
Cardiac arrhythmias	59 (9.12%)	44 (5.06%)	0.054	44 (8.10%)	39 (7.18%)	0.568
Chronic pulmonary disease	111 (17.16%)	104 (11.95%)	0.004	84 (15.47%)	88 (16.21%)	0.740
Liver disease	13 (2.01%)	19 (2.18%)	0.815	11 (2.03%)	11 (2.03%)	> 0.999
Renal failure	101 (15.61%)	92 (9.12%)	0.004	70 (12.89%)	75 (13.81%)	0.656
Coagulopathy	65 (10.05%)	63 (7.24%)	0.052	50 (9.21%)	43 (7.92%)	0.448
ECI	0.00 (0.00–15.00)	0.00 (0.00–11.00)	< 0.001	4.00 (0.00–12.00)	3.00 (0.00–12.00)	0.147
*Vital signs*						
HR, beats/min	84.03 ± 15.31	81.06 ± 15.00	< 0.001	83.91 ± 14.83	81.81 ± 15.66	0.029
SBP, mmHg	113.16 ± 16.46	113.41 ± 15.03	0.758	113.96 ± 16.53	113.15 ± 15.61	0.400
DBP, mmHg	59.41 ± 10.36	60.97 ± 9.68	0.001	59.83 ± 10.18	59.93 ± 9.73	0.855
MBP, mmHg	77.24 ± 10.54	78.07 ± 9.58	0.111	77.97 ± 10.45	77.41 ± 9.62	0.338
RR, times/min	19.27 ± 3.86	18.33 ± 3.28	< 0.001	19.22 ± 3.82	18.64 ± 3.46	0.009
Temperature, ℃	36.85 ± 0.71	36.80 ± 0.62	0.127	36.87 ± 0.68	36.78 ± 0.69	0.030
SpO_2_, %	97.04 ± 2.44	97.32 ± 2.72	0.039	97.19 ± 2.18	97.09 ± 3.24	0.541
*Laboratory parameters*						
WBC, 10^9^/L	13.97 ± 6.37	12.11 ± 5.33	< 0.001	13.11 ± 5.08	13.30 ± 6.00	0.520
PLT, 10^9^/L	240.35 ± 103.55	226.43 ± 95.44	0.007	238.25 ± 103.67	230.50 ± 89.77	0.177
LMR	1.88 (1.29–2.40)	4.84 (3.81–6.50)	< 0.001	1.93 (1.36–2.44)	4.75 (3.68–6.36)	< 0.001
Hb, g/dL	11.47 ± 2.18	11.68 ± 2.11	0.059	11.58 ± 2.18	11.63 ± 2.16	0.692
HCT, %	33.85 ± 6.34	34.18 ± 6.02	0.304	34.07 ± 6.33	34.16 ± 6.19	0.816
Glu, mg/dL	174.64 ± 91.80	164.88 ± 87.53	0.036	172.76 ± 91.90	176.47 ± 99.44	0.533
Scr, mg/dL	1.10 (0.90–1.70)	0.90 (0.80–1.30)	< 0.001	1.00 (0.80–1.40)	1.00 (0.80–1.40)	0.977
BUN, mg/dL	24.00 (17.00–35.00)	17.00 (13.00–25.00)	< 0.001	22.00 (16.00–30.00)	21.00 (15.00–29.00)	0.421
*Scoring system*						
SIRS	3.00 (2.00–4.00)	3.00 (2.00–3.00)	< 0.001	3.00 (2.00–4.00)	3.00 (2.00–4.00)	0.035
SAPSII	37.00 (28.00–48.00)	30.00 (23.00–41.00)	< 0.001	35.00 (27.00–44.00)	35.00 (26.00–47.00)	0.465
SOFA	4.00 (2.00–7.00)	2.00 (1.00–5.00)	< 0.001	3.00 (2.00–6.00)	3.00 (1.00–6.00)	0.628
*Treatment information*						
PCI	296 (45.75%)	491 (56.44%)	< 0.001	267 (49.17%)	287 (52.85%)	0.225
CABG	86 (13.29%)	173 (19.89%)	0.001	81 (14.92%)	101 (18.60%)	0.104

Data are presented as mean ± SD, median (interquartile range), or number of patients (%)

*PSM* propensity score matching, *BMI* body mass index, *ECI* elixhauser comorbidity index, *HR* heart rate, *SBP* systolic blood pressure, *DBP* diastolic blood pressure, *MBP* mean blood pressure, *RR* respiratory rate, SpO_2_, percutaneous oxygen saturation; *WBC* white blood cell, *PLT* platelet, *LMR* lymphocyte-to-monocyte ratio, *Hb* hemoglobin;, *HCT* hematocrit, *Glu* glucose, *Scr* serum creatinine, *BUN* blood urea nitrogen, *SIRS* systemic inflammatory response syndrome, *SAPS* simplified acute physiology score, *SOFA* sequential organ failure assessment, *PCI* percutaneous transluminal coronary intervention, *CABG* coronary artery bypass grafting

### Outcomes

Patients in low LMR group had longer ICU length of stay (3.12 vs. 2.08 days, *P* < 0.001) and hospital length of stay (7.42 vs. 5.17 days *P* < 0.001) compared to high LMR group before PSM. Notably, the low LMR group had a high risk of hospital mortality (19.78 vs. 10.34%, *P* < 0.001), 30-day mortality (22.10 vs. 11.84%, *P* < 0.001) and 1-year mortality (36.32 vs. 19.20%, *P* < 0.001). After matching, ICU length of stay (3.01 vs. 2.38 days, *P* < 0.001), hospital length of stay (7.09 vs. 5.44 days *P* < 0.001) and 1-year mortality (32.04 vs. 26.15%, *P* < 0.001) remained significantly different between the two groups, while no differences were observed in hospital mortality (16.21 vs. 14.18%, *P* = 0.352) and 30-day mortality (18.60 vs. 16.02%, *P* = 0.261) (Table 2).

**Table 2:** Outcomes of patients before and after PSM matched

	LMR < 3.00	LMR ≥ 3.00	*P* value
Before PSM	N = 647	N = 870	
ICU length of stay, days	3.12 (1.62–7.06)	2.08 (1.24–3.90)	< 0.001
Hospital length of stay, days	7.42 (4.05–13.89)	5.17 (3.21–9.23)	< 0.001
Hospital mortality, n (%)	128 (19.78%)	90 (10.34%)	< 0.001
30-day mortality, n (%)	143 (22.10%)	103 (11.84%)	< 0.001
1-year mortality, n (%)	235 (36.32%)	167 (19.20%)	< 0.001

After PSM	N = 543	N = 543	
ICU length of stay, days	3.01 (1.55–6.85)	2.38 (1.32–4.78)	< 0.001
Hospital length of stay, days	7.09 (4.03–13.68)	5.44 (3.24–10.08)	< 0.001
Hospital mortality, n (%)	88 (16.21%)	77 (14.18%)	0.352
30-day mortality, n (%)	101 (18.60%)	87 (16.02%)	0.261
1-year mortality, n (%)	174 (32.04%)	142 (26.15%)	0.033

*LMR* lymphocyte-to-monocyte ratio, *PSM* propensity score matching

### Survival analysis

**Fig. 1:**
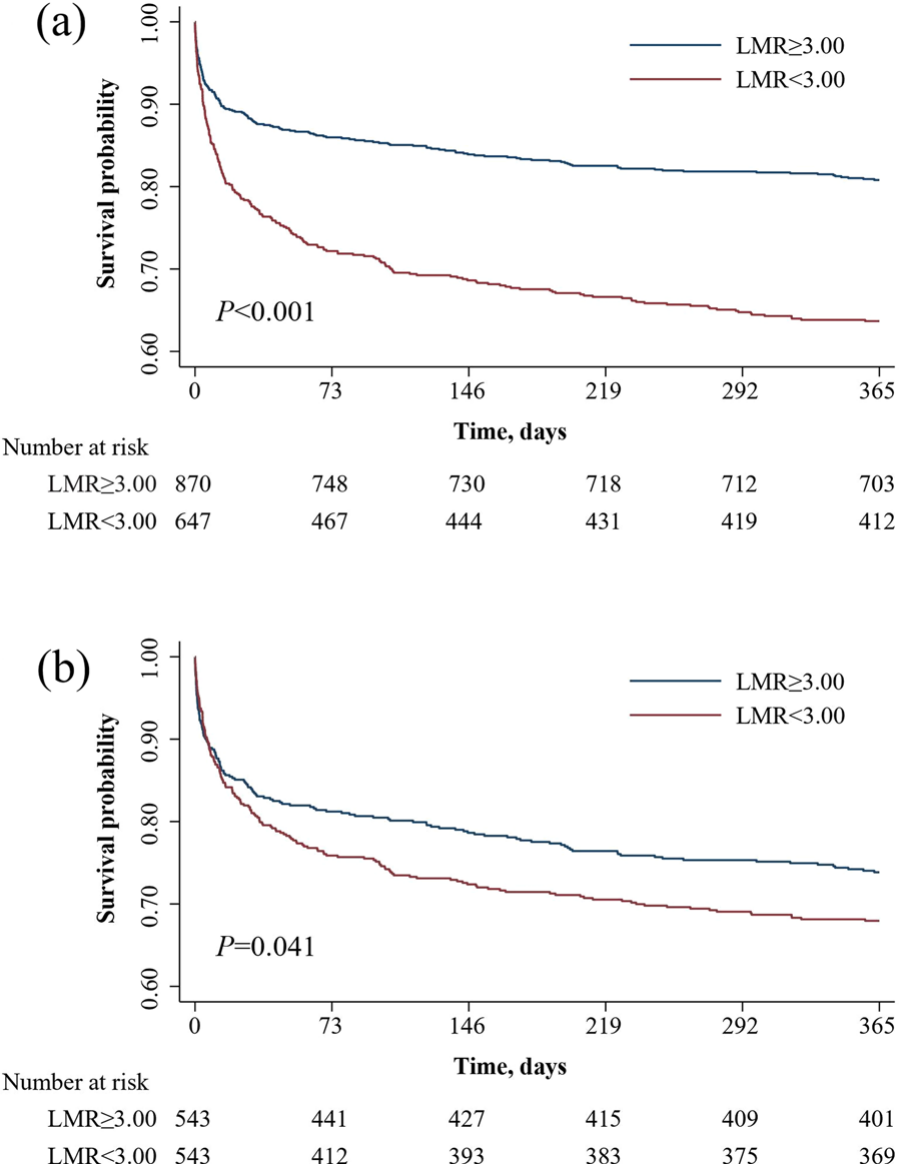
Kaplan–Meier survival analysis plot for 1-year survival in the high LMR group vs. low LMR group before **a** and after **b** propensity score matching. *LMR* lymphocyte-to-monocyte ratio

The survival curves for patients of different LMR groups were shown in a Kaplan–Meier plot in Fig. 1. Patients with low LMR had a significant lower 1-year survival rate compared to high LMR group whether before (63.83 vs.81.03%, Log rank *P* < 0.001) or after (68.13 vs.74.22%, Log rank *P* = 0.041) PSM.

A Cox regression model was performed to determine the association between LMR and 1-year mortality of AMI patients. Variables with *P* values less than 0.05 in the univariate Cox regression analysis were included to be adjusted in model II for multivariate analysis, while model I was only adjusted by age, gender and ethnicity. The multivariate analysis showed that low LMR was associated with increased risk of 1-year mortality compared to high LMR (Model I: HR = 2.060, 95%CI 1.688–2.515, *P* < 0.001; Model II: HR = 1.369, 95%CI 1.110–1.687, *P* = 0.003) (Table 3). After matching, the higher risk of 1-year mortality remained significant in low LMR group (Model I: HR = 1.279, 95%CI 1.024–1.598, *P* = 0.030; Model II: HR = 1.299, 95%CI 1.032–1.634, *P* = 0.026) (Table 4).

**Table 3:** Univariate and multivariate Cox regression analysis for 1-year mortality before PSM

	Univariate analysis			Multivariate analysis					
				Model I			Model II		
	HR	95%CI	*P*	HR	95%CI	*P*	HR	95%CI	*P*
LMR < 3.00	2.094	1.717–2.554	0.000	2.060	1.688–2.515	0.000	1.369	1.110–1.687	0.003
Age, years	1.004	1.002–1.005	0.000	1.003	1.002–1.004	0.000	1.002	1.001–1.003	0.004
Gender, Male	0.673	0.553–0.819	0.000	0.727	0.592–0.892	0.002	0.945	0.758–1.178	0.614
Ethnicity, White	0.739	0.605–0.902	0.003	0.733	0.600–0.896	0.002	0.760	0.618–0.935	0.010
Hypertension	0.680	0.556–0.831	0.000				1.077	0.859–1.351	0.519
Congestive heart failure	1.978	1.526–2.563	0.000				0.760	0.568–1.018	0.066
Cardiac arrhythmias	1.970	1.449–2.679	0.000				1.315	0.938–1.843	0.113
Chronic pulmonary disease	1.339	1.036–1.731	0.026				1.027	0.787–1.341	0.844
Renal failure	1.800	1.407–2.302	0.000				1.159	0.859–1.562	0.334
Coagulopathy	2.012	1.523–2.658	0.000				1.104	0.816–1.494	0.521
HR, beats/min	1.022	1.016–1.028	0.000				1.006	0.999–1.014	0.091
DBP, mmHg	0.964	0.955–0.975	0.000				0.991	0.979–1.002	0.124
RR, times/min	1.100	1.073–1.128	0.000				1.041	1.014–1.070	0.003
SpO_2_, %	0.915	0.895–0.935	0.000				0.948	0.927–0.969	0.000
WBC, 10^9^/L	1.046	1.033–1.059	0.000				1.000	0.985–1.015	0.992
PLT, 10^9^/L	1.000	0.999–1.001	0.833				–	–	–
Hb, g/dL	0.867	0.829–0.907	0.000				0.957	0.905–1.012	0.126
Glu, mg/dL	1.003	1.002–1.003	0.000				1.001	1.000–1.002	0.013
Scr, mg/dL	1.193	1.143–1.245	0.000				1.060	0.968–1.159	0.208
BUN, mg/dL	1.019	1.016–1.022	0.000				0.999	0.992–1.005	0.643
SIRS	1.506	1.360–1.669	0.000				1.069	0.940–1.216	0.307
SAPSII	1.058	1.052–1.064	0.000				1.045	1.034–1.056	0.000
SOFA	1.228	1.199–1.259	0.000				0.997	0.952–1.045	0.914
PCI	0.526	0.430–0.643	0.000				0.695	0.551–0.878	0.002
CABG	0.405	0.285–0.575	0.000				0.394	0.266–0.582	0.000

*PSM* propensity score matching, *LMR* lymphocyte-to-monocyte ratio, *HR* heart rate, *DBP* diastolic blood pressure, *RR* respiratory rate, SpO2, percutaneous oxygen saturation; *WBC* white blood cell, *PLT* platelet, *Hb* hemoglobin, *Glu* glucose, *Scr* serum creatinine, *BUN* blood urea nitrogen, *SIRS* systemic inflammatory response syndrome, *SAPS* simplified acute physiology score, *SOFA* Sequential organ failure assessment, *PCI* percutaneous transluminal coronary intervention, *CABG* coronary artery bypass grafting

**Table 4:** Univariate and multivariate Cox regression analysis for 1-year mortality after PSM

	Univariate analysis			Multivariate analysis					
				Model I			Model II		
	HR	95%CI	*P*	HR	95%CI	*P*	HR	95%CI	*P*
LMR < 3.00	1.260	1.009–1.572	0.041	1.279	1.024–1.598	0.030	1.299	1.032–1.634	0.026
Age, years	1.033	1.024–1.042	0.000	1.033	1.023–1.043	0.000	1.025	1.014–1.036	0.000
Gender, Male	0.719	0.575–0.898	0.004	0.933	0.738–1.180	0.564	1.096	0.855–1.405	0.470
Ethnicity, White	0.648	0.517–0.811	0.000	0.642	0.513–0.805	0.000	0.651	0.515–0.823	0.000
Hypertension	0.629	0.501–0.791	0.000				0.888	0.688–1.146	0.360
Congestive heart failure	1.612	1.188–2.186	0.002				0.876	0.620–1.236	0.451
Cardiac arrhythmias	1.567	1.097–2.236	0.013				0.926	0.624–1.374	0.702
Chronic pulmonary disease	1.167	0.874–1.557	0.295				–	–	–
Renal failure	1.580	1.192–2.093	0.001				0.908	0.638–1.292	0.591
Coagulopathy	2.088	1.532–2.848	0.000				1.325	0.944–1.861	0.104
HR, beats/min	1.020	1.013–1.027	0.000				1.009	1.001–1.018	0.034
DBP, mmHg	0.970	0.959–0.982	0.000				0.998	0.984–1.011	0.726
RR, times/min	1.087	1.057–1.118	0.000				1.053	1.022–1.085	0.001
SpO_2_, %	0.928	0.902–0.954	0.000				0.953	0.930–0.977	0.000
WBC, 10^9^/L	1.024	1.005–1.043	0.012				1.003	0.982–1.024	0.805
PLT, 10^9^/L	0.999	0.998–1.001	0.299				–	–	–
Hb, g/dL	0.870	0.827–0.915	0.000				0.962	0.903–1.024	0.223
Glu, mg/dL	1.002	1.002–1.003	0.000				1.001	1.000–1.002	0.060
Scr, mg/dL	1.173	1.116–1.233	0.000				1.090	0.989–1.201	0.082
BUN, mg/dL	1.021	1.017–1.026	0.000				0.997	0.989–1.004	0.389
SIRS	1.372	1.220–1.542	0.000				0.937	0.804–1.092	0.405
SAPSII	1.056	1.049–1.063	0.000				1.034	1.021–1.047	0.000
SOFA	1.222	1.187–1.257	0.000				1.067	1.010–1.126	0.020
PCI	0.637	0.509–0.796	0.000				0.721	0.559–0.930	0.012
CABG	0.433	0.294–0.639	0.000				0.414	0.270–0.633	0.000

*PSM* propensity score matching, *LMR* lymphocyte-to-monocyte ratio, *HR* heart rate, *DBP* diastolic blood pressure, *RR* respiratory rate, SpO2, percutaneous oxygen saturation; *WBC* white blood cell, *PLT* platelet, *Hb* hemoglobin, *Glu* glucose, *Scr* serum creatinine, *BUN* blood urea nitrogen, *SIRS* systemic inflammatory response syndrome, *SAPS* simplified acute physiology score, *SOFA* sequential organ failure assessment, *PCI* percutaneous transluminal coronary intervention, *CABG* coronary artery bypass grafting

### Subgroup analysis

**Fig. 2:**
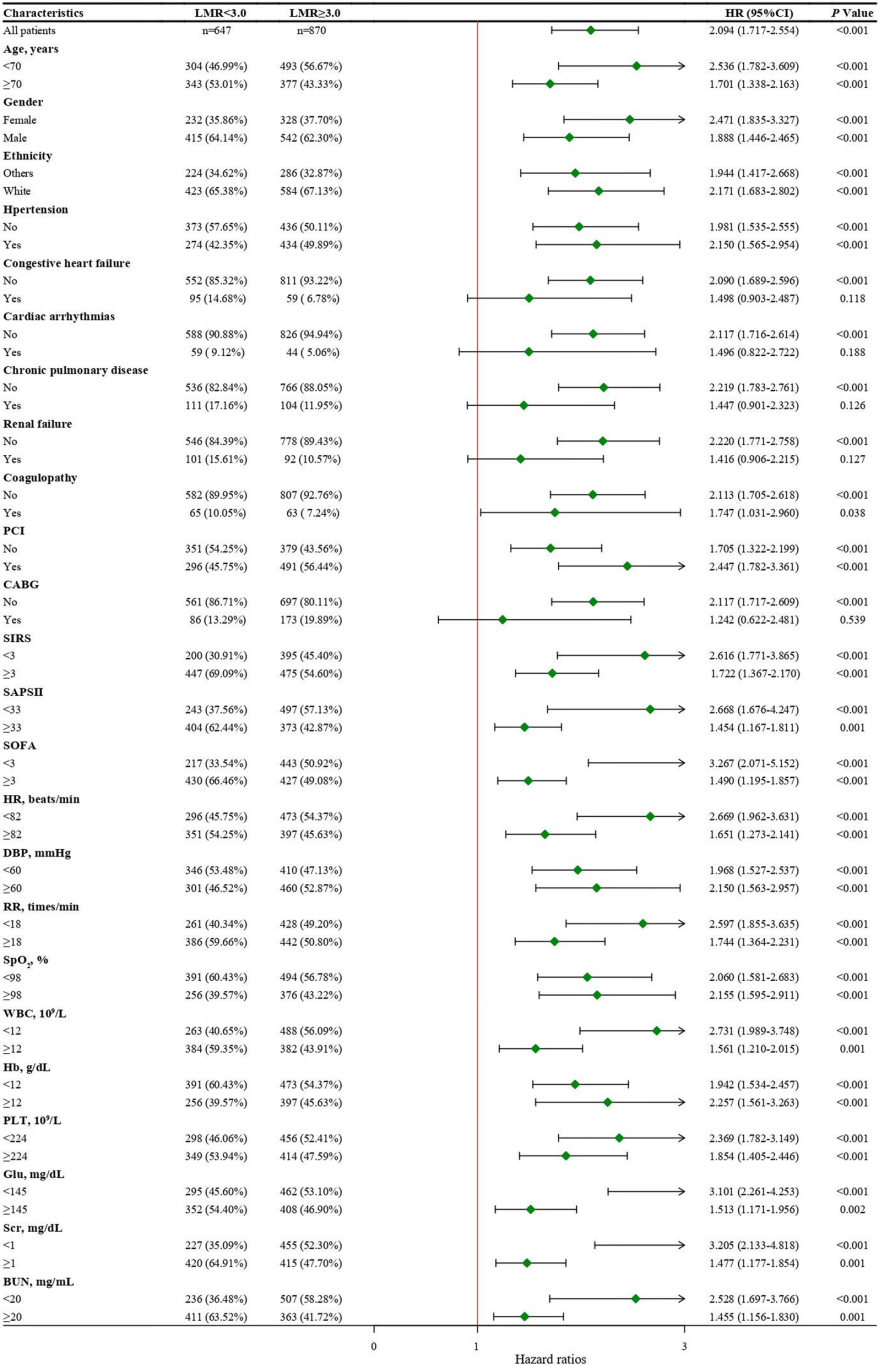
Association between LMR group and 1-year mortality of AMI patients in different subgroups. *LMR* lymphocyte-to-monocyte ratio, *PCI* percutaneous transluminal coronary intervention, *CABG* coronary artery bypass grafting, *SIRS* systemic inflammatory response syndrome, *SAPS* simplified acute physiology score, *SOFA* sequential organ failure assessment, *HR* heart rate, *DBP* diastolic blood pressure, *RR* respiratory rate, SpO_2_, percutaneous oxygen saturation; *WBC* white blood cell, *PLT* platelet, *Hb* hemoglobin, *Glu* glucose, *Scr* serum creatinine, *BUN* blood urea nitrogen

Subgroup analysis of variables with significant differences in baseline characteristics was performed to verify the stability of the Cox regression results. As shown in the Fig. 2, AMI patients with an LMR < 3.00 had higher risk of 1-year mortality than those with an LMR ≥ 3.00 in subgroups except for the patients with congestive heart failure (HR = 1.498, 95%CI 0.903–2.487, *P* = 0.118), cardiac arrhythmias (HR = 1.496, 95%CI 0.822–2.722, *P* = 0.188), chronic pulmonary disease (HR = 1.447, 95%CI 0.901–2.323, *P* = 0.126), renal failure (HR = 1.416, 95%CI: 1.416, 95%CI 0.906–2.215, *P* = 0.127) and CABG treatment (HR = 1.242, 95%CI 0.622–2.481, *P* = 0.539).

## Discussion

Circulating leukocyte subtype counts is considered to be of great value for disease evaluation and prognosis prediction of inflammatory injury patients. In the present study, we demonstrated an independent relationship between admission LMR and 1-year mortality in AMI patients. To our knowledge, this is the first study focusing on the association between the measurement of LMR and long-term prognosis of critically ill AMI patients.

Acute myocardial infarction, usually caused by plaque rupture and interruption of coronary blood flow, triggers an intense inflammatory response which is essential for myocardium repair. Alternatively, hyperactivity and prolonged inflammation after infarction may lead to myocardial dysfunction [25]. The critical role of immune cells in the pathophysiological process after myocardial infarction is now being deeply revealed [4]. Each component of immune cells plays a dynamic role in pro-inflammation response and anti-inflammation repair stage after AMI [3, 26].

As indicators of systemic inflammatory status, the role of WBC and its subtypes in the diagnosis, risk stratification and prognostic prediction of AMI has also been demonstrated in various clinical trials and practices [9–11, 27]. In previous studies, both a low lymphocyte counts and high monocyte counts were associated with an increased risk for major adverse cardiovascular events [28, 29]. Although the role of macrophages in inflammatory response after IHD has been widely recognized [6], the potential mechanisms of low lymphocyte level and its predictive value are not fully understood. Acute lymphocytopenia is generally considered to be part of the stress response and is associated with increased cortisol and sympathetic activation [30]. The increased lymphocytes apoptosis could also explain the association with adverse outcomes [31].

Unlike simple cell counts, ratios between different WBC subtypes, such as NLR, can fully combine the prognostic information of different components to provide greater predictive abilities [9]. LMR, as a novel hematologic indicator which is calculated by dividing lymphocyte count by monocyte count, has shown great prognostic value in cardiovascular diseases such as heart failure [19] and acute coronary syndrome [20, 21, 32–34]. To further investigate the relationship between LMR and long-term prognosis in critically ill AMI patients, we constructed a retrospective cohort in MIMIC-III database using cut-off values generated by X-tile software, and showed that low admission LMR levels were independently associated with higher risk for 1-year mortality.

In this study we performed PSM analysis, which helps to balance confounding factors in baseline characteristics. Although there were no significant differences in hospital mortality and 30-day mortality between the LMR groups after PSM, the main outcomes we focused on remained consistent before (36.32% vs. 19.20%, *P* < 0.001) and after matching (32.04% vs. 26.15%, *P* = 0.033). The HRs of 1-year mortality with an LMR < 3.00 were changed before and after PSM (Model I:2.060 vs. 1.279; Model II:1.369 vs. 1.299), which may be the result of the equilibrium of baseline characteristics, or related to the change of optimal cutoff values after PSM. Furthermore, procedure events during hospitalization were also included in the Cox regression model. The results showed that PCI and CABG were both protective factors for 1-year mortality before and after matching, suggesting that MI patients may benefit from aggressive coronary revascularization, which is consistent with the recommendations of guideline [35]. Due to the relatively small sample size and single-center-based cohort of the current study, further studies based on larger populations with external validation are warranted.

To validate the robustness of the regression results, subgroup analysis which containing variable with significant differences in baseline characteristics was performed to examine the statistical potency of LMR under different conditions. LMR maintained its predictive ability in most subgroups except in patients with CHF, cardiac arrhythmias, chronic pulmonary disease, renal failure, or CABG treatment. On the one hand, underlying disease such as heart failure usually suggest worse pathophysiological conditions and thus interfere with the long-term prognosis of LMR. On the other hand, CABG treatment which is a powerful protection factors shown in Cox regression (HR = 0.394, 95%CI 0.266–0.582, *P* < 0.001) could balance the mortality between two groups. Generally, LMR demonstrated its excellent predictive value and stability in our research.

Several limitations of our study should be noted. Firstly, patients with myocardial infarction were identified using ICD-9 codes rather than clinical diagnostic criteria, and few patients were inevitably ignored. Secondly, the information of admission WBC subtype was missing in some patients. Therefore, they were excluded from our case cohort, which may lead to selection bias. Thirdly, since our sample size is relatively small and the cohort is single-center, the possibility that the optimal cut-off value may vary with different study populations. Fourthly, factors such as hematological, inflammatory and infectious diseases that may affect the counts of circulating immune cells were not excluded as exclusion criteria because of the difficulty in obtaining accurate and detailed information from the database. Further studies and external validation based on large multicenter prospective cohorts are needed to determine the most appropriate LMR cut-off value for different populations.

## Conclusions

In this retrospective cohort analysis, we demonstrated that a low admission LMR (< 3.00) was associated with a higher risk of 1-year mortality in critical ill AMI patients. Our findings provide an affordable, convenient, and reliable tool for clinical prediction of long-term adverse outcomes in MI patients.

## Supplementary Information

**Additional file 1**. **Figure S1**: Flow chart: the inclusion of the study population. *ICU* intensive care units, *AMI* acute myocardial infarction. **Figure S2**: Propensity score matching graph between two LMR groups. *LMR* lymphocyte-to-monocyte ratio.

## Data Availability

The datasets used and analysed during the current study are available from the corresponding author on reasonable request.

## Abbreviations

AMI: Acute myocardial infarction
LMR: Lymphocyte-to-monocyte ratio
MIMIC-III: Medical Information Mart for Intensive Care III
ICUs: Intensive care units
PSM: Propensity score matching
CVDs: Cardiovascular diseases
IHD: Ischemic heart disease
WBC: White blood cells
PLR: Platelet to lymphocyte ratio
NLR: Neutrophil to lymphocyte ratio
ICD-9: International Classification of Diseases, Ninth Revision
BMI: Body mass index
HR: Heart rate
SBP: Systolic blood pressure
DBP: Diastolic blood pressure
RR: Respiratory rate
SpO_2_: Percutaneous oxygen saturation
CHF: Congestive heart failure
ECI: Elixhauser comorbidity index
PLT: Platelet count
Hb: Hemoglobin
HCT: Hematocrit
Glu: Glucose
BUN: Blood urea nitrogen
Scr: Serum creatinine
SIRS: Systemic inflammatory response syndrome
SAPS: Simplified acute physiology score
SOFA: Sequential organ failure assessment
PCI: Percutaneous coronary intervention
CABG: Coronary bypass artery grafting
