# Supplementary material for: The prognostic value of admission lymphocyte-to-monocyte ratio in critically ill patients with acute myocardial infarction
Source: BMC Cardiovasc Disord. 2022 Jul 7;22:308. doi: 10.1186/s12872-022-02745-z (PMC9264617; doi:10.1186/s12872-022-02745-z)
Supplement: Supplementary file 1 — Additional file 1. Figure S1: Flow chart: the inclusion of the study population. ICU intensive care units, AMI acute myocardial infarction. Figure S2: Propensity score matching graph between two LMR groups. LMR lymphocyte-to-monocyte ratio. [file 12872_2022_2745_MOESM1_ESM.docx]

Supplementary Material

## Supplementary Figures


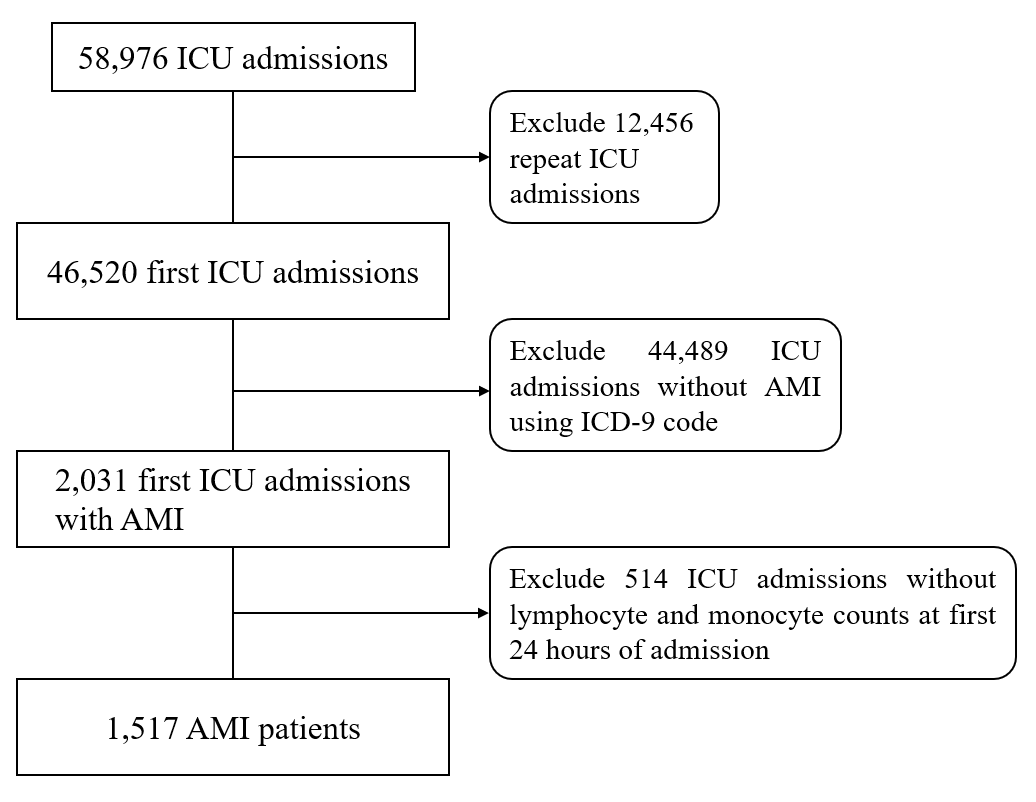


**Supplementary Figure 1.** Flow chart: the inclusion of the study population. Abbreviations: ICU, intensive care units; AMI, acute myocardial infarction.


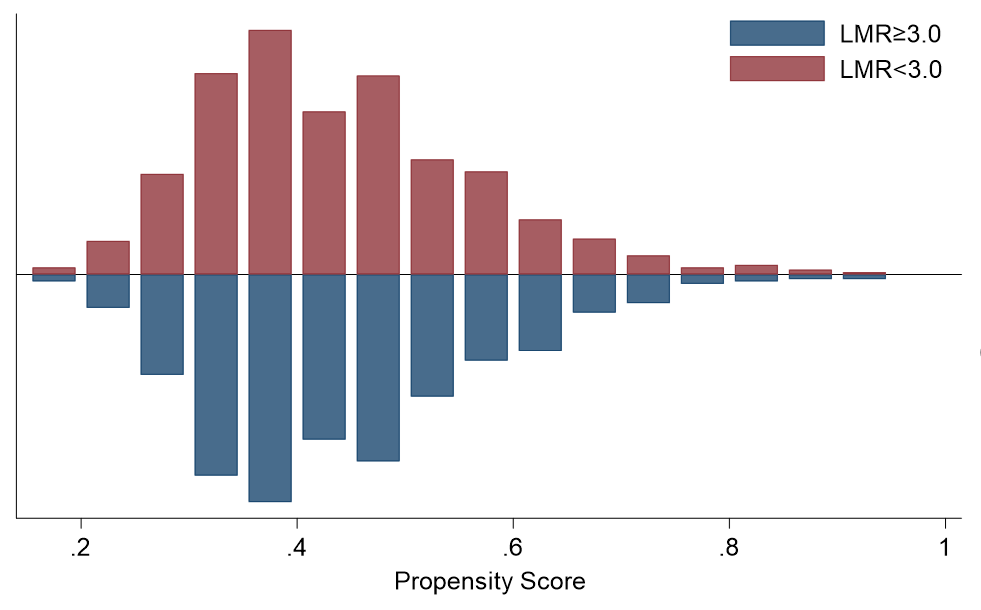


**Supplementary Figure 2.** Propensity score matching graph between two LMR groups. Abbreviations: LMR: lymphocyte-to-monocyte ratio.
